# General practitioners not available – out-of-hospital emergency patients handled by anaesthesiologist in a large Norwegian municipality

**DOI:** 10.1080/02813432.2021.1922833

**Published:** 2021-06-07

**Authors:** Henrik Hauståker, Øyvind Østerås, Dag Ståle Nystøyl, Jon Kenneth Heltne, Erik Zakariassen

**Affiliations:** a Faculty of Medicine, University of Bergen, Bergen, Norway; b Department of Anaesthesia and Intensive Care, Haukeland University Hospital, Bergen, Norway; c Department of Global Public Health and Primary Care, University of Bergen, Bergen, Norway; d Department of Research, Norwegian Air Ambulance Foundation, Drøbak, Norway; e Faculty of Medicine, Department of Clinical Medicine, University of Bergen, Bergen, Norway; f Health Services Research Group, Department of Global Public Health and Primary Care, University of Bergen, Bergen, Norway; g National Centre for Emergency Primary Health Care, NORCE Health, Bergen, Norway

## Abstract

**Background:**

Until autumn 2018 the GPs in Bergen Municipality did not attend emergency patients outside the emergency primary care centre. The ambulance staff handled emergencies on their own or were assisted by an anaesthesiologist from the helicopter emergency medical service (HEMS). The aim of this study was to investigate procedures performed by the HEMS anaesthesiologist and to assess the level of skills needed to perform these procedures.

**Methods:**

This study was a retrospective assessment of data from the period 2011 to 2013 on all emergency missions in which patients were dealt with by HEMS, using a rapid-response car in Bergen Municipality. All emergency missions were sorted into three categories: No intervention, Basic or Advanced intervention. This list was made by a research group with anaesthesiologists working for Bergen HEMS and GPs with OOH experience. The list is based on curriculum found in acute medicine courses.

**Results:**

HEMS responded to 716 (2.3%) out of a total of 31,696 emergencies in Bergen Municipality during the three years. In more than two-thirds (71%) of these missions, no intervention or only a basic intervention was performed. Most advanced procedures were performed in patients with cardiac arrest.

**Conclusion:**

By retrospective evaluation of HEMS missions by car in Bergen municipality, we found that nearly one-third of the patients received advanced procedures. Cardiac arrest was the medical condition in which the most advanced procedures were performed. More research is needed to evaluate procedures and the importance of clinical evaluation and physicians’ experience in treating these patient groups.

**KEY POINTS**
- Both HEMS and on-call GPs are needed in emergency care, and more knowledge will be useful to highlight the level of practical skills needed in these missions.
- There is a need for better prioritization of when to use HEMS resources and when to use on-call GPs in emergency missions.
- More than two-thirds of the patients involved in emergency missions received no intervention or just a basic intervention when dealt with by HEMS.
- This raises the issue of whether an on-call GP could have adequately treated many of the patients in this study in terms of practical skills.

## Introduction

Prehospital emergency medical services in Norway include Emergency Medical Communication Centres (EMCCs), ambulance services (ground, boat and air) and on-call general practitioners (GPs) as part of the out-of-hours (OOH) service. The specialist healthcare system is responsible for the EMCCs and the ambulance service. The municipalities are responsible for on-call GPs and the OOH emergency primary healthcare service [1]. The municipalities must provide an emergency system that responds to the population’s need for immediate help, and that ensures that a GP is available. The on-call GPs must, when necessary, perform a call-out to the patient site in the event of accidents and other emergencies, provide assistance and decide on the level of care needed [1]. Trained nurses at the EMCCs use the Norwegian Index of Medical Emergencies to classify medical problems in accordance with one of three different levels of response: red (emergency), yellow (urgent) and green (normal) [2]. A red response indicates an immediate need for help, and the EMCC will simultaneously send an alarm to the ambulances and to on-call GPs in the relevant geographical area [1,2].

The helicopter emergency medical system (HEMS) is staffed with an experienced anaesthesiologist, a rescue paramedic and a pilot [1], and is primarily used in the event of illness or trauma requiring rapid transportation, advanced assessment/triage or advanced medical treatment. In addition to the helicopter, HEMS has a rapid-response car with medical equipment similar to that used in the helicopters for call-outs in nearby areas or when helicopter evacuation is not available. When alarmed, HEMS chooses whether to use a rapid-response car or a helicopter. The criteria for alerting the anaesthesiologist-manned rapid response car is the same as for use of a helicopter, and thus shall not replace alerting on-call GP at the OOH service [3].

Previous studies from Norway have shown that the ambulance staff appreciates having a GP on site when responding to critically ill patients [4] and that GPs take part in and improve patient care in emergency missions in cooperation with the ambulance staff [4–6]. In Europe, GPs are involved in responses involving emergency patients, but the approach differs from country to country [7,8]. As an example, GPs in Denmark to a large extent provide telephone triage, home visits and give advice [7], whilst anaesthesiologists attend emergency patients outside the hospitals [9], using a wide-ranging and well-developed system of rapid-response cars and helicopters.

In Norway the healthcare system is built around the ‘LEON’ principle (the principle of the Lowest Efficient Care Level), to ensure healthcare at the right level is administered by the right healthcare personnel. This principle states that primary healthcare, and not the specialist health service, should take care of the investigation, treatment and follow-up that can be performed better or equally well at this level of care [10]. The benefits of prehospital critical care are generally accepted. Different emergency services do different procedures in prehospital emergencies, due to different levels of expertise [11]. A systematic review concludes that HEMS crew tended to be more experienced and have a larger ability to perform advanced procedures, compared to ground-based crew [12]. One of the challenges in research on the topic is large differences between the types of services being compared [13]. As a first step to investigate if the GP could handle patients in cooperation with the ground ambulance the issue of emergency procedures performed by the anaesthesiologist is important, due to the Norwegian emergency system with both a GP and an anaesthesiologist on call. To better understand what kinds of emergency patients lie within the area of expertise of an on-call GP, more knowledge is needed regarding the types of procedures actually performed in medical emergencies, and the level of skills needed to perform these procedures. Until autumn 2018 the GPs in Bergen Municipality did not attend emergency patients outside the emergency primary care centre [14,15]. In these cases, the ambulance staff needed to handle the situation on their own. In potentially serious emergency situations, when there has been a need for medical expertise above the ambulance level, the HEMS anaesthesiologist has been the only assistance available. This background makes Bergen Municipality a suitable study object.

The aim of this study was thus to investigate procedures performed by the HEMS anaesthesiologist in rapid-response car missions in Bergen Municipality and to assess the level of skills needed to perform these procedures.

## Material and methods

Every HEMS mission in Bergen is registered in the ‘Airdoc’ database. The data includes administrative and time data, the patient’s vital signs and the treatment provided, and there is also a free-text field. We performed a retrospective cross-sectional study of all mission data from 1st Jan 2011 to 31st Dec 2013. All the available emergency missions in which the HEMS Bergen anaesthesiologists dealt with patients using the rapid-response car in Bergen Municipality were included. In 2015 there were 250,420 people living in the 86 km^2^ area of Bergen Municipality, and thus 2915 inhabitants per km^2^ [16].

The research group, comprising anaesthesiologists working for Bergen HEMS and GPs with OOH experience, developed a list of procedures within the expected area of expertise of a GP and an anaesthesiologist with experience in prehospital emergency medicine (Table 1). The list is divided into two parts: one including procedures within both the on-call GPs’ and the anaesthesiologists’ area of expertise (basic), and another only involving procedures within the anaesthesiologists’ area of expertise (advanced). The list of procedures characterized as ‘basic procedures’ is made up accordingly to the curriculum found in acute medicine courses for GPs on-call in Norway, and the National Centre for Emergency Primary Health Care in Norway. Based on this, all the red responses by HEMS were sorted into three categories: No intervention, Basic or Advanced intervention. No intervention by an anaesthesiologist refers to the cases where the anaesthesiologist dealt with the patient but did not initiate any intervention. The data included from the records comprised gender, age, alarm time, NACA scores, a tentative diagnosis, procedures performed and treatment provided. All 716 cases were categorised into ten medical conditions based on the prehospital medical diagnoses made by the anaesthesiologist. The anaesthesiologist selected the condition most likely to be the patient’s true medical problem. Drowning, cardiac arrest caused by trauma, foreign-body airway obstruction and all external impacts causing injury were classified as trauma [17,18].

**Table 1.** List of basic and advanced procedures.

Basic procedures	Advanced procedures
Examination Basic airway manoeuvres Relieve pressure pneumothorax with needle Stabilising and splinting fractures Stopping external bleeding with compression / elevation / packing / tourniquet Blood glucose measurement and management Use of supraglottic airway device Rhythm analysis and defibrillation Pain treatment Intravenous fluid treatment Prehospital thrombolysis Mouth-to-mouth ventilation and use of pocket mask Inhalation therapy Mask / bag ventilation and assisted ventilation Oxygen treatment Chest compressions Establish intravenous access Establish intraosseous access Immobilisation of trauma patient with use of splinting device (e.g. SAM sling) Use of CPAP ECG Treatment of seizures and overdoses	Blood-product administration Cricothyrotomy Use of ultrasound Induction and maintenance of anaesthesia Use of ketamine, fentanyl and suxamethonium chloride Endotracheal intubation and monitoring of intubated patient during transport Thoracic drainage Establishing CVC access Establishing invasive blood-pressure measurement Respirator treatment

Norway’s air-ambulance service uses the National Committee on Aeronautics (NACA) Score to classify the severity of a patient’s medical problem [19]. Based on the available information, the anaesthesiologist classifies the patient’s status from 0 to 7, where 0 indicates no injury or disease and 7 indicates that the patient is dead. A NACA score between 0 and 3 indicates a non-life-threatening situation, whilst a NACA score of 4 or higher indicates a possibly life-threatening situation. The highest NACA score during the mission was registered.

### Ethics and approval

The Regional Ethics Committee (REK Vest 2010/2930) examined the protocol for the study and waived the need for approval. The Ministry of Health and Care Services (2011–02407), the Data Protection Officials for Research and the Norwegian Data Protection Authority (12/00291–3) approved the study.

### Statistical analyses

The Statistical Package for the Social Sciences (SPSS) Version 24 (IBM Corp., Armonk, NY, USA) was used for descriptive statistical methods. Continuous data are presented as mean and SD for normally distributed data, and median and IQR for skewed data. The number of red responses in Bergen Municipality is presented as rates per 1000 inhabitants per year with 95% CI.

## Results

During the study period, 31,696 red-response missions in Bergen Municipality were registered. This represents an average of 10,565 red-response situations per year in Bergen, that is, 42 (CI 41.4–44.0) red-response cases per 1000 inhabitants per year. The anaesthesiologist responded to 716 (2.3%) of all the 31,696 missions, and at least one procedure was performed on 480 (67%) of the patients attended.

Nearly half of the 716 patients had a potentially life-threatening condition, whilst a fifth of all the patients died. Most procedures, both basic and advanced, were performed on patients with a NACA score above 3 (Table 2). In most of the missions, no procedure or only a basic procedure was performed by HEMS. In the patients in need of advanced procedures (206), 86 (42%) were patients with a NACA score of 7.

**Table 2.** NACA distribution and procedures performed by the anaesthesiologists.

Procedures								
	None		Basic		Advanced		Total	
NACA	*n*	%	*n*	%	*n*	%	*n*	%
0–3	149	60	96	39	3	1	248	100
4–6	56	17	152	47	117	36	325	100
7	31	22	26	18	86	60	143	100
Total	236	33	274	38	206	29	716	100

Of all the 1197 procedures performed by HEMS, a fifth were defined as advanced (Table 3). The anaesthesiologist performed between zero and nine procedures on each patient. Within the advanced intervention group, over 50% of the procedures were intubations (Table 3). Of all the 716 cases, 220 (31%) patients suffered a cardiac arrest and 218 (31%) trauma (Table 4). In the patients in need of advanced procedures (206), 149 (72%) procedures were performed in patients with cardiac arrest and 38 (18%) in trauma patients. The majority of the patients with a potentially life-threatening condition had a medical problem.

**Table 3.** A list of all procedures initiated by the anaesthesiologist during the study period.

Intervention level	Procedure	Frequency	%
Advanced	Intubation	125	11
	Advanced drug therapy	51	4
	Arterial-catheter access	15	1
	Central venous-catheter access	11	1
	Other^a^	33	3
	Total	235	20
Basic	Basic drug therapy	241	20
	Fluid treatment	141	12
	Oxygen treatment	136	11
	Peripheral venous-catheter access	96	8
	Orogastric tube	52	4
	12-lead ECG	40	3
	Assisted ventilation	39	3
	Intraosseous needle access	35	3
	Use of neck collar	34	3
	CPR	34	3
	Defibrillation	33	3
	Immobilisation of the patient	23	2
	Other^b^	58	5
	Total	962	80
All procedures	Total number of procedures	1197	100

^a^Refers to other advanced procedures such as thorax drainage, use of blood products, anaesthesia; ^b^refers to other basic procedures such as measurement of blood glucose, immobilisation of trauma patient, use of CPAP: prehospital thrombolysis.

**Table 4.** Interventions by the anaesthesiologist and medical conditions

Medical condition	Intervention class						Total	
	No intervention		Basic intervention		Advanced intervention			
	*n*	%	*n*	%	*n*	%	*n*	%
Cardiac Arrest	29	12	42	15	149	72	220	31
Trauma	93	39	87	32	38	18	218	31
Breathing difficulties	12	5	21	8	4	2	37	5
Chest pain	2	1	14	5	1	1	17	2
Stroke	3	1	2	1	4	2	9	1
Acute neurology, e.g. stroke	32	14	42	15	6	3	80	11
Psychiatry and intoxication	19	8	19	7	1	1	39	6
Obstetrics and childbirth	11	5	1	0	1	0	13	2
Infection	12	5	12	4	0	0	24	3
Other*	23	10	34	13	2	1	59	8
Total	236	100	274	100	206	100	716	100

*'Other' refers to all the diagnoses that cannot be allocated to any of the nine other diagnosis categories, e.g. arrhythmia, anaphylaxis, gastrointestinal bleeding.

## Discussion

The anaesthesiologist responded to 716 of all 31,696 red response missions in the period. In most of the missions, no procedure or only a basic procedure was performed by HEMS. Advanced procedures were needed in 206 patients, and cardiac arrest constituted the majority.

### Strengths and limitations

This study was a cross-sectional study involving all the available data from 2011 through to 2013. Our study provides useful information on all registered missions, which includes procedures, over a period of three years. The population and the prehospital services have not changed significantly between 2013 and recently in Bergen Municipality. However, as of autumn 2018, the on-call GP in Bergen Municipality has attended emergency patients on site using a rapid-response car, and this could decrease the number of missions for HEMS anaesthesiologists.

Clinical evaluation is difficult to measure and was not part of the scope of this study. This list of procedures only differentiates between the different assignments based on emergency medical procedures. It is also possible that not all procedures were registered since the data was not originally collected for research purposes. One could also argue that the list of basic procedures is rather comprehensive. Is it realistic that GPs on-call masters them all? This will remain unclear, but the list is in accordance with the curriculum found in acute medicine courses for GPs on-call in Norway, and the National Centre for Emergency Primary Health Care in Norway. Based on this most GPs should be familiar with most of the basic procedures. Still, we do want to highlight that what is listed in a curriculum may not correspond to the actual knowledge in the relevant area in a daily clinical practise, nor that the actual skill is mastered by every GP’s on call. However, a previous study showed that GPs in Norway probably could perform more than half of the procedures initiated by the HEMS anaesthesiologist on route to the hospital [20]. We also want to highlight that there is a limitation in dividing procedures into a list without taking into account the context the procedure often occurs in. For example, mask/bag ventilation, establishing a supraglottic airway and reliving pneumothorax by the needle are considered basic procedures. But it often happens in a context with patients in need of critical care medicine in need of other treatment. Therefore, the procedure itself may be basic, but the context may be ‘advanced’. The list of procedures will not highlight this aspect.

Another limitation of this study is that it lacks formal validation of whether advanced treatment was really needed when provided. Since 42% of the advanced procedures were performed in patients with a NACA score of 7, one could argue that these procedures did not have an effect. Further, we found that most advanced procedures were carried out in cardiac arrest patients, but only a small portion of these received CPR and defibrillation, according to the registration. CPR was in most cases already started by first responders or the ambulance personnel, not started by the HEMS. The study also lacks information about the patient outcome after hospital treatment. Application of the results in this study to other areas lacking GP involvement in out-of-hospital emergencies must be carried out with caution.

### Comparison with previous studies

We found an average of 10,565 red-response situations per year in Bergen, that is, 42 emergency responses per 1000 inhabitants per year. The average rate of emergency responses in Norway has been between 21/1000 and 27/1000 inhabitants/year [2,18,21]. The reason for the high rate of emergency-response missions found in Bergen Municipality is unknown, though urban variables such as drug abuse and overdose, as well as a large number of schools, students and working people, may explain it. However, there are reports from Norway’s larger EMCCs of an increase in red-response missions in recent years [18].

Nine out of ten of the advanced procedures performed by HEMS were in patients suffering from cardiac arrest or trauma, and the most frequent advanced procedures were intubation and advanced drug therapy. Based on this finding, and excluding cardiac arrest, HEMS only performed advanced procedures in 11% of the emergency missions. A study from Denmark found that the treatment provided was defined as being lifesaving in 2.7% of all call-outs, and that the anaesthesiologist performed procedures that went beyond the expertise of the attending ambulance staff in 85% of the cases [22]. One difference between the studies is that the Danish study divided the missions into two groups, namely ‘within the area of expertise of EMTs/paramedics’ and ‘in need of an anaesthesiologist’, whilst our study also had the group ‘within the area of expertise of a GP’. This might explain why we found that a third of the missions carried out procedures that require an anaesthesiologist, whilst the Danish study found that 85% of the missions required an anaesthesiologist.

In our study 248 of the 716 patients (34.6%) had a NACA score of between 0 and 3. This might be a result of an overtriage and may partly explain the high rate of no procedures and basic procedures carried out by the anaesthesiologist. However, overtriage to emergency missions are expected and, in many cases, due to sparse patient information. Trauma patients are often young, and injuries may affect the long-term outcome, even with NACA score lower than 4. By removing the NACA 0–3 group, we find that advanced procedures were performed in 203 of the 468 (43%) missions, and that ‘no intervention started by the anaesthesiologist’ only accounts for 87 of the 468 (19%) missions (Table 2). It is also important to note that the reason for the anaesthesiologist not carrying out procedures may be a result of the ambulance staff already having performed the necessary procedures before the anaesthesiologist arrived, and of the anaesthesiologist having monitored the effect of the procedures already carried out. It is also important to highlight that having a HEMS anaesthesiologist present, creates security if the need for an advanced procedure should arise, even if performing an advanced procedure or not, one is prepared if needed. Therefore, in retrospect, many missions might be performed by GP-manned services. However, in several missions, an anaesthesiologist would be advantageous, in performing advanced procedures and in advanced clinical assessment. Our message is that a GP service in the municipality may reduce the number of missions for the anaesthesiologist-manned rapid response car, not replace the anaesthesiologist. This required good triage at the EMCC, which often is a challenge [23].

A study from 2013 described all air-ambulance dispatches in Western Norway and found that in approximately two-thirds of the primary missions the patient had a NACA score of between 4 and 7, and that advanced procedures were performed in 41% of all missions [18]. In a comparison with our data, the NACA score between 4 and 7 is almost the same, but advanced procedures only account for 29% of the cases in our material. This might be a result of the short distance to highly specialised hospital-treatment facilities in Bergen. In most cases in Bergen, and for most rapid-response car missions, the appropriate decision is most often to initiate rapid transportation to the hospital and avoid performing time-consuming medical procedures on site. Thus, fewer procedures may be provided in Bergen Municipality than in areas further away from a hospital. In addition, avoiding advanced intervention is not the same as providing no advanced clinical assessment. Choosing not to perform advanced interventions could sometimes be an advanced medical decision [21]. The current study only investigated emergency patients for whom the anaesthesiologist chose to use the rapid-response car within Bergen Municipality. One can thus argue that if a hospital is close by, the right call will in most cases be to load and go, rather than spending time on advanced prehospital procedures. This may partly explain why we are reporting less use of advanced procedures than other studies [18]. Another assumption could be that the anaesthesiologists performed missions for emergency patients that on-call GPs in other municipalities handle alone.

Since the ambulance staff took care of patients without any physician in nearly all emergency responses in Bergen Municipality during the period 2011–2013, we might well ask whether there is any need for a prehospital physician in this municipality. Trained ambulance personnel do perform basic emergency procedures, and therefore one could ask if there is any benefit of a physician-staffed emergency medical service. However, the absence of a GP in the event of an emergency might contribute to unnecessary transportation of patients straight to the emergency department, as shown in a previous study [24]. Also, the list of procedures separating an anaesthesiologist’s expertise from that of an on-call GP included neither an anaesthesiologist’s nor a GP’s clinical evaluation of the situation, nor experience of emergency-medicine situations nor the quality of the clinical examination [6]. The importance of sound clinical evaluations in emergencies is difficult to measure, though in some situations it might be time-saving if an experienced physician triaged the patient to the right level of care and treatment. Advanced procedures performed by an experienced anaesthesiologist or GP may primarily improve patient physiology and reduce patient discomfort. However, the ability to decide if or when to perform an advanced procedure may be more difficult. Based on the findings regarding procedures alone, GPs could be able to relieve HEMS anaesthesiologists in some of the emergency missions, and this will probably be beneficial since the capacity of HEMS is limited [6,18]. However, choosing which emergency missions are suitable for GPs alone, without the need for an anaesthesiologist, is difficult.

## Conclusion

By retrospective evaluation of HEMS missions by car in Bergen municipality, we found that during a period without GPs available, and with anaesthesiologists handling prehospital emergency patients, that one-third of the patients received no procedures, one third received basic procedures and nearly one-third advanced procedures. Cardiac arrest was the medical condition in which the most advanced procedures were performed by the attending anaesthesiologist. More research is needed to evaluate not only procedures but also the importance of clinical evaluation and physicians’ experience in treating these patient groups. Together with research in triage criteria to distinguish between different levels of care needed, this may optimize prehospital care and use of resources.
